# Survivin Promotes Piperlongumine Resistance in Ovarian Cancer

**DOI:** 10.3389/fonc.2019.01345

**Published:** 2019-11-29

**Authors:** Xing-Wei Nan, Li-Hua Gong, Xu Chen, Hai-Hong Zhou, Piao-Piao Ye, Yang Yang, Zi-Hao Xing, Meng-Ning Wei, Yao Li, Sheng-Te Wang, Kun Liu, Zhi Shi, Xiao-Jian Yan

**Affiliations:** ^1^Department of Gynecology, The First Affiliated Hospital of Wenzhou Medical University, Wenzhou, China; ^2^Guangdong Provincial Key Laboratory of Bioengineering Medicine, Department of Cell Biology and Institute of Biomedicine, National Engineering Research Center of Genetic Medicine, College of Life Science and Technology, Jinan University, Guangzhou, China; ^3^Center for Uterine Cancer Diagnosis & Therapy Research of Zhejiang Province, Women's Hospital and Institute of Translational Medicine, Zhejiang University School of Medicine, Hangzhou, China

**Keywords:** piperlongumine, survivin, ovarian cancer, ROS, proteasome

## Abstract

Ovarian cancer is one of the most fatal female malignancies while targeting apoptosis is critical for improving ovarian cancer patients' lives. Survivin is regarded as the most robust anti-apoptosis protein, and its overexpression in ovarian cancer is related to poor survival and apoptosis resistance. Piperlongumine (PL) extracted from peppers is defined as an active alkaloid/amide and exhibits a broad spectrum of antitumor effects. Here, we demonstrate that PL induces the rapid depletion of survivin protein levels via reactive oxygen species (ROS)-mediated proteasome-dependent pathway *in vitro*, while exerting a remarkable inhibitory influence on the proliferation of ovarian cancer cells. Overexpression of survivin raises the survival rate of ovarian cancer cells to PL. Moreover, PL inhibits ovarian cancer cells xenograft tumor growth and downregulates survivin *in vivo*. Our findings reveal a previously unrecognized mechanism of PL in suppressing survivin expression as well as survivin promotes piperlongumine resistance in ovarian cancer and suggest that ROS-mediated proteasome-dependent pathway can be exploited to overcome apoptosis resistance triggered by aberrant expression of survivin.

## Introduction

Ovarian cancer is the most fatal female reproductive tract cancer and the seventh most common cancer among women all over the world (1). It was evaluated that 22,240 ovarian cancer cases would be diagnosed while concurrently 14,070 women would succumb to the disease in 2018 (2). Between 1976 and 2015, owing to effective approaches to the clinical treatment, including aggressive cytoreductive surgery followed by system chemotherapy, the mortality rate of ovarian cancer declined by 33% (2). However, despite the markable achievement of ovarian cancer during the last decade, the prognosis for women with advanced disease remains poor and chemoresistance appears to be a significant obstacle constraining the clinical application (3). Progress in curbing the incidence and mortality of human ovarian cancer can be propelled by the development of novel agents.

Survivin, one of the inhibitor of apoptosis proteins family (IAP) firstly found in 1997, may accelerate the progress of cancer via promoting the insurgence of mutations as well as inducing resistance to chemotherapy (4). While undetectable in terminally differentiated adult tissues, the survivin gene has been proved to be overexpressed in lots of cancers including ovarian cancer (5) in which survivin overexpression is detected in 74% of case and is related to the advanced clinical stage (6). Furthermore, our previous studies showed that the cytoplasmic protein expression level of survivin was an independent prognosis marker for ovarian cancer, downregulating survivin expression could enhance apoptosis (7, 8). Thus, survivin could be a promising target for apoptosis-based treatment in ovarian cancer therapy (9).

Piperlongumine (PL) extracted from the long pepper (*Piperlongum L*.) belongs to a biological alkaloid, embodying potential selective cytotoxic and antitumor properties over cancer cells of several histo-types including ovarian cancer (10). Our previous studies firstly demonstrated that PL facilitated G2/M cell cycle arrest, impeded the proliferation of human ovarian cancer cells, and induced reactive oxygen species (ROS) mediated apoptosis (11). Our previous cell-based experiments (11) and data published (12) indicated that the effect of PL to suppress the growth of tumors without general toxicity and to perturb redox and ROS homeostasis verify the PL treatment as a potential therapeutic opportunity against ovarian cancer.

However, the detailed molecular mechanism of the PL-mediated antitumor effect is not well-understood and still requires further investigation. Here, we firstly demonstrate that PL depleted the expression of survivin protein via a ROS-mediated proteasome-dependent pathway in ovarian cancer cells. We further evaluate the efficacy of PL to inhibit tumor growth *in vivo* as a potentially effective therapy for ovarian cancer treatment.

## Materials and Methods

### Cell Culture

The cancer cell lines A2780, OVCAR-3, and HEK293T were cultured in DMEM (Gibco, NY, USA). Ten percent fetal bovine serum (Gibco, NY, USA) and antibiotics (10 mg/mL streptomycin and 10,000 U/mL penicillin) were additionally supplemented. The aforementioned cell lines were cultivated in a 37°C incubator at 5% CO_2_.

### Chemicals and Antibodies

PL, N-Acetyl-cysteine (NAC) and Chloroquine (CQ) were obtained from Sigma Chemical Co (St. Louis, MO, USA). MG132 was from ApexBio. The HRP secondary antibodies, anti-survivin (#2808), and anti-PARP (#9542) were obtained from Cell Signaling Technologies (Danvers, MA, USA). Anti-Vinculin (BM1611) antibody was obtained from Boster, China. Anti-β-actin (KM9001), and anti-GAPDH (KM9002) antibodies were obtained from Tianjin Sungene Biotech Co., China. MG132, CQ, and NAC were added 1 h before PL treatment in all co-treated experimented.

### Western Blot

Cells were lysed in RIRA buffer at 4°C for around 30 min followed by centrifugation at 13,200 × rpm for 10 min to remove nuclei and other cell debris. Total protein concentration was detected by the Micro BCA Protein Assay Kit (Sangon Biotech, C503061) and the lysates were either used immediately or stored at −80°C. 10–12% SDS-PAGE gels were used to separate the proteins extracted before and thereby separated proteins were completely transferred to polyvinylidene difluoride (PVDF) membranes. Five percent BSA was used to block the membranes for 1 h and the indicated primary antibodies were added and incubated overnight. Membranes were probed with the chemiluminescent detection reagents, and reactive bands were visualized using UVP ChemStudio PLUS (Analytikjena) (13, 14).

### RT-PCR

Total RNA was extracted from 5 × 10^6^ to 5 × 10^7^ cells using the RaPure Total RNA Mini Kit (Magen, #R4011-02), treated with DNAseI to eliminate genomic DNA, and quantitated using the Epoch spectrophotometer. Reverse transcription (RT) followed instructions provided by StarScript II First-strand cDNA Synthesis Kit-II (GenStar, #A214-05). The resulting cDNA was used as a template for the amplification of target gene transcripts by RT-PCR, using Hieff^TM^ qPCR SYBR^®^ Green Master Mix (YEASEN, #11201ES08) on the Hema9600 PCR machine. After 35 amplification cycles, reaction products were analyzed and β-actin RNA was used as a loading control. The primer sequences were as follows: survivin forward: CCGACGTTGCCCCCTGC; survivin reverse: TCGATGGCACGGCGCAC; β-actin forward: AAATCGTGCGTGACATTAAGC; β-actin reverse: CCGATCCACACGGAGTACTT (15, 16).

### Lentivirus Production and Infection

pDONR201-survivin was inserted into pCDH-Neo-Venus/DEST via LR clonase (Invitrogen, #11791) to form pCDH-Neo-Venus-survivin plasmid. At a 4:3:1 ratio, the lentiviral transfer vector, packaging plasmids psPAX2 and pMD.2G were transferred to the HEK293T cells to produced lentivirus. The PEI reagent was performed for transfection. Then, the viral supernatant was, respectively, collected 24, 48, 72, 96 h following transfection, filtered through a 0.20 μm filter, and concentrated. Using the polybrene (Solarbio, H8761), A2780, OVCAR-3 cells were transfected with pCDH-Neo-Venus-survivin or pCDH-Neo-Venus/DEST, followed by incubation with 48 h for the subsequent trials (17, 18).

### Cell Viability Assay

It was assessed using a 3-(4,5-dimethylthiazol-2-yl)-2,5-diphenyltetrazolium bromide (MTT) assay. Briefly, ovarian cancer cells were seeded in 96-well plates, 4,500 cells per well, supplementing with 100 μL of medium and exposed to PL at different concentrations for 72 h, followed by the addition of 10 μL of MTT solution (5 mg/mL) per well to the medium and incubation 4 h at 37°C. After we discarded the medium, 50 μL of DMSO per well was used to elute the blue MTT-formazan product, and absorbance of the solution was read at 570 nm (19, 20).

### Nude Mice Xenograft Assay

Balb/c nude female mice with 4–5 weeks of age and 20–22 g weight were purchased from the Guangdong Medical Laboratory Animal Center. For *in vivo* experiments, 4 × 10^6^ A2780 cells in 100 μL of medium were injected subcutaneously into the left and right shoulders of each mouse. After the subcutaneous tumors reached a size of 0.3 × 0.3 cm^2^, mice were randomized to treatment with PL (20 mg/kg) intraperitoneally or 0.5% methylcellulose alone every day. In the end, the tumors were got rid of mice and weighed after they were sacrificed. The bodyweight and the tumor volume (V) of mice were recorded every day. Formula: V = π/6 (1/2 (A + B))^3^, the rate of inhibition (IR) = 1 – Mean tumor weight of experiment group/Mean tumor weight of control group × 100% (21, 22).

### Statistical Analysis

Statistical significance of differences was determined by student's *t*-test. Differences were defined as significant at *P* < 0.05.

## Results

### PL Downregulates the Expression of Survivin in Ovarian Cancer Cells

**Figure 1:**
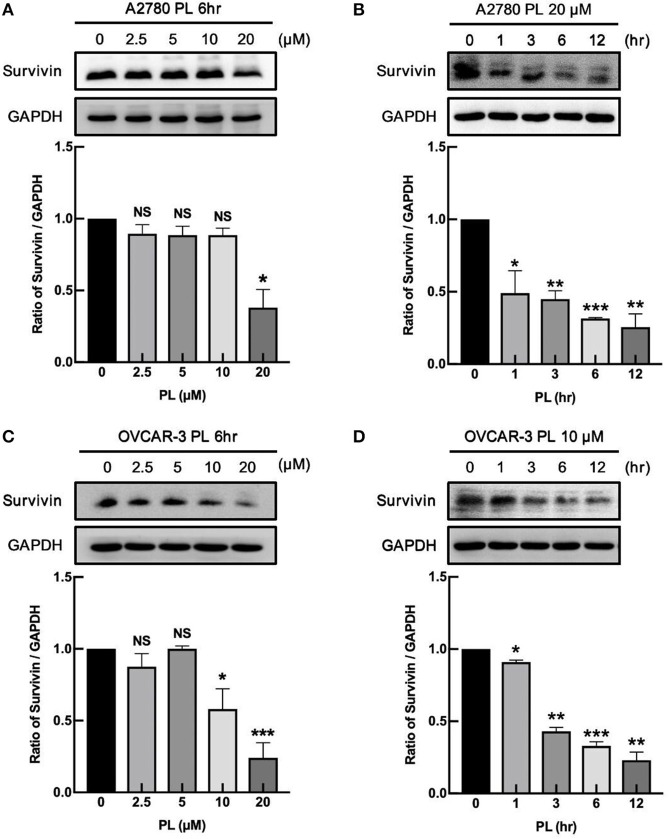
PL downregulates the expression of survivin in ovarian cancer cells. A2780 **(A)** and OVCAR-3 **(C)** cells were treated with PL for indicated periods with different concentrations (0, 2.5, 5, 10, 20 μM), respectively. A2780 **(B)** and OVCAR-3 **(D)** cells were treated with 20 or 10 μM PL for 0, 1, 3, 6, 12 h. The expression of survivin was assayed by immunoblotting. GAPDH was used as a loading control. The band quantification was performed using ImageJ software. The representative of three experiments. ^*^P < 0.05, ^**^P < 0.01, and ^***^P < 0.001, Student's *t*-test. NS, not significant.

**Figure 2:**
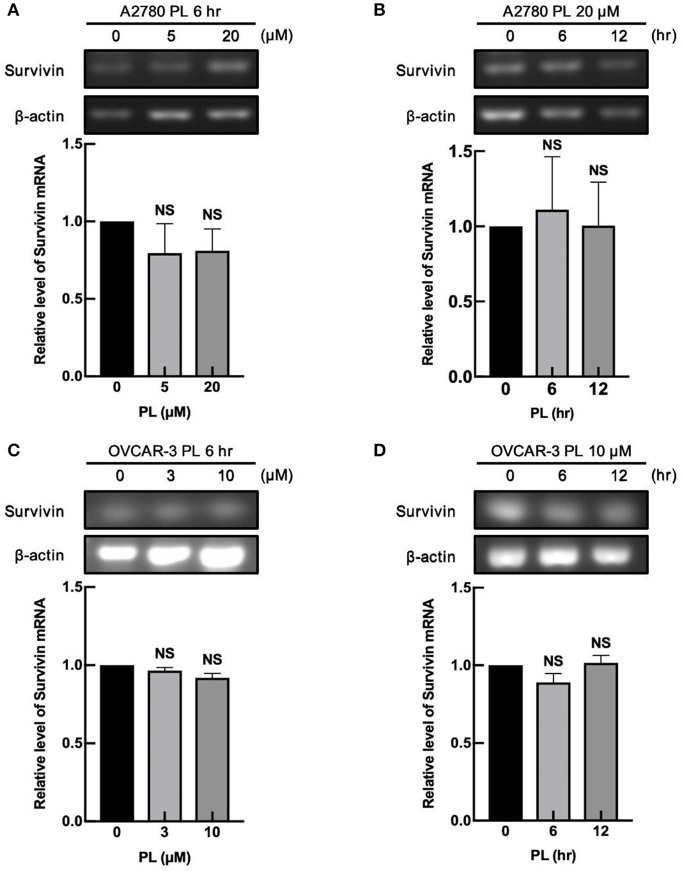
PL does not alter mRNA expression of survivin. A2780 **(A)** and OVCAR-3 **(C)** cells were treated with indicated concentrations of PL for 6 h. Moreover, A2780 **(B)** and OVCAR-3 **(D)** cells were treated with 20 or 10 μM PL for the indicated periods. Survivin mRNA levels were detected by RT-PCR using specific primers. β-actin served as a loading control. The band quantification was performed using ImageJ software. The representative of three experiments. Student's *t*-test. NS, not significant.

Our previous study firstly demonstrated that PL facilitated G2/M cell cycle arrest, impeded the proliferation of human ovarian cancer cells, and induced ROS dependent apoptosis in ovarian cancer (11). Due to the ability to inhibit the cell apoptosis, survivin may take part in various processes associated with the tumor development and progression, facilitated metastasis and angiogenesis, as well as favor cell cycle progression (9). Because of the apoptotic effect of PL induced in ovarian cancer cells, we explored the negative regulation of PL on survivin expression. As shown in **Figures 2A,C**, A2780 and OVCAR-3 cells were exposed to increasing concentrations of PL (up to 20 μM) for 6 h, and the survivin protein was detected by western blot assay. PL effectively depleted the survivin expression in both A2780 and OVCAR-3 cells at the low micromolar concentration (10 or 20 μM) in contrast to the control cells. Furthermore, as demonstrated in Figures 1B,D, downregulation of survivin was significant as soon as 6 h following PL treatment. Together, these evidences manifested that PL induces the reduction of survivin ovarian cancer cells *in vitro*, which may contribute to its apoptotic effect.

### PL Does Not Alter mRNA Expression of Survivin

To deliberate the discipline of PL-mediated survivin reduction, our group detected whether PL regulates the survivin mRNA expression in ovarian cancer cells. A2780 and OVCAR-3 cells were exposed to increasing concentrations of PL for 6 h or treated for different time scales at the same concentration of PL (10 or 20 μM), and the expression levels of survivin mRNA were examined by RT-PCR. In Figures 2A–D, the survivin mRNA expression levels were not altered both on concentration and time scales, which were not similar to the depletion of survivin protein levels. These findings strongly supported the notion that the PL regulates survivin gene expression at the non-transcriptional level.

### PL Induces Proteasome-Dependently Degradation of Survivin

**Figure 3:**
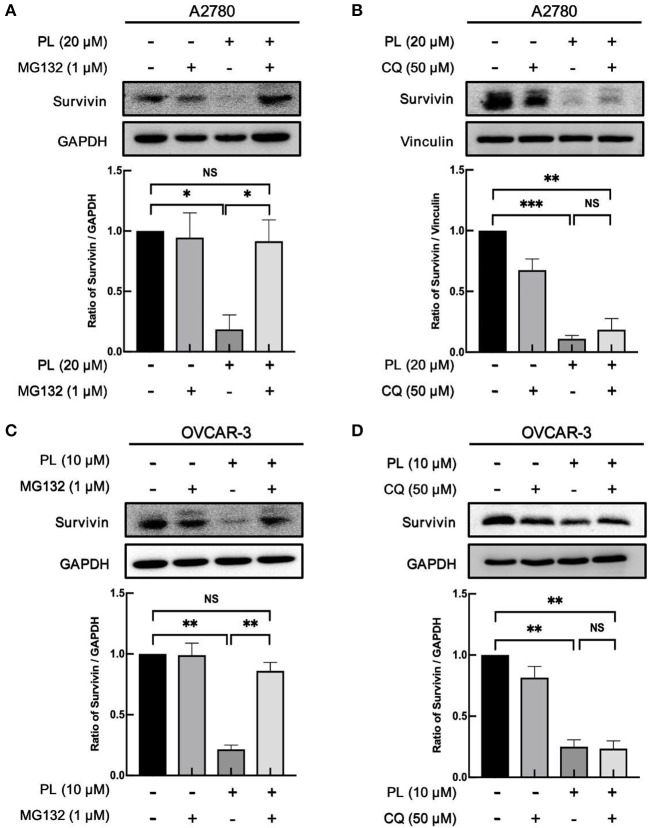
PL induces proteasome-dependently degradation of survivin. A2780 **(A)** and OVCAR-3 **(C)** cells were pre-treated with 1 μM MG132 for 1 h and cultured with the indicated concentration of PL for 24 h. A2780 **(B)** and OVCAR-3 **(D)** cells were pre-treated with 50 μM CQ for 1 h and cultured with the indicated concentration of PL for 24 h. The expression of survivin was detected by immunoblotting. GAPDH and Vinculin were used as loading controls. The band quantification was performed using ImageJ software. Results were obtained based on three separate experiments. ^*^P < 0.05, ^**^P < 0.01, and ^***^P < 0.001, Student's *t*-test. NS, not significant.

The aforementioned findings indicated that PL might induce depletion of survivin at the post-transcription level. The possible discipline expounding PL-induced decrease of survivin protein expression may associate with its rapidly increased ubiquitin-proteasome degradation. In order to verify this supposition, our group preincubated A2780 and OVCAR-3 cells with proteasome inhibitors MG132 (1 μM). The results presented in Figures 3A,C demonstrated that MG132 reversed PL-mediated survivin depletion compared with control cells, hinting that PL-mediated degradation of survivin occurs via ubiquitin-proteasome mechanism.

Otherwise, the autophagy-lysosome system and the ubiquitin-proteasome system are the essential intracellular proteolytic pathways in eukaryotes (23). To clarify whether the degradation of survivin protein was related to the induction of autophagy, CQ, a potent inhibitor of autophagy, was used to verify this hypothesis. However, in contrast to the result of the MG132, the expression levels of survivin stayed unchanged in response to autophagy inhibition (Figures 3B,D), indicating that the cellular autophagic flux is not involved in the PL-mediated degradation of survivin expression. Collectively, these results showed that PL induces the depletion of survivin via the proteasome-dependent pathway.

### PL Induces ROS-Dependently Apoptosis and Survivin Depletion

**Figure 4:**
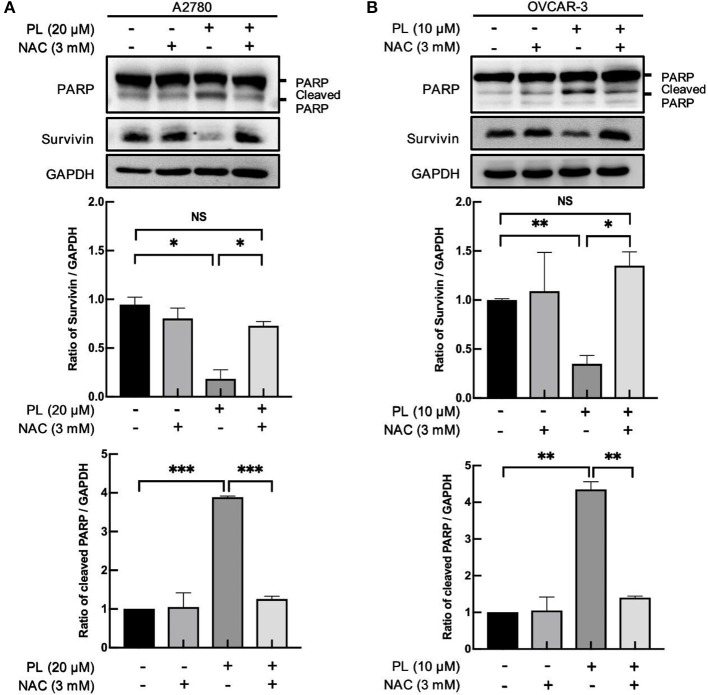
PL induces ROS-dependently apoptosis and survivin depletion. A2780 **(A)** and OVCAR-3 **(B)** cells were treated with the indicated concentration of PL for 24 h in the present or absent of 3 mM NAC pretreatment for 1 h. Western blotting was taken to detect PARP, cleaved PARP, and survivin protein levels. GAPDH served as a loading control. The band quantification was performed using ImageJ software. Results were obtained based on three separate experiments. ^*^P < 0.05, ^**^P < 0.01, and ^***^P < 0.001, Student's *t*-test. NS, not significant.

Lots of anticancer agents perform the antitumor property by the activation of intracellular ROS. Indeed, it has been elucidated that PL-induced apoptotic cell death in mammary tumors and sarcoma are closely related to the increased elevation of the intracellular ROS level (24). Of note, our previous study found that PL caused a prominent rise in ROS levels and induced significant apoptosis in ovarian cancer cells (11). Meanwhile, co-administration of PL and NAC fully restored the PL-mediated increase in ROS and inhibited the depletion of survivin (Figures 4A,B). Taken together, these data offered additional support to our hypothesis that ROS generation is significant for PL-mediated ovarian cancer cells apoptosis and survivin degradation.

### Overexpression of Survivin Raises the Survival Rate of Ovarian Cancer Cells to PL

**Figure 5:**
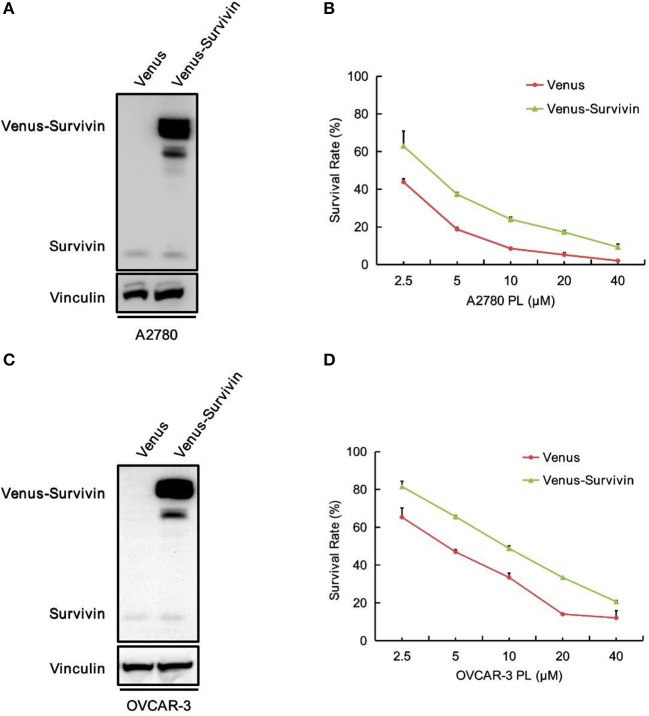
Overexpression of survivin raises the survival rate of ovarian cancer cells to PL.A2780 **(A)** and OVCAR-3 **(C)** cells were infected with lentivirus for overexpressing survivin. Protein levels were examined by Western blot. Vinculin was used as a loading control. The viability of A2780 **(B)** and OVCAR-3 **(D)** cells were measured by MTT assay.

To explore the role of survivin involved in PL-induced ovarian cancer apoptosis, we performed survivin infection taking advantage of pCDH-Neo-Venus-survivin lentivirus, while pCDH-Neo-Venus/DEST was adopted as the control. The Western blotting analysis was used to confirm the result of infection. As shown in Figures 5A,C, A2780 and OVCAR-3 cells infected with pCDH-Neo-Venus-survivin exhibited a much higher survivin expression. To detect the role of survivin in the apoptosis effect of PL, stable cells overexpressing survivin were treated with the increasing concentrations of PL and assayed to examine the survival rate (Figures 5B,D). In the presence of PL, the IC_50_ value of OVCAR-3 cells was 4.59 μM. However, in survivin overexpression cells, the IC_50_ value was 9.65 μM, which was 2.10-fold higher than the control group. Consistently, similar results were also demonstrated in A2780 cells. Collectively, these results suggested that survivin overexpression raises the survival rate of ovarian cancer cells.

### PL Inhibits A2780 Xenograft Tumor Growth and Downregulates Survivin *in vivo*

**Figure 6:**
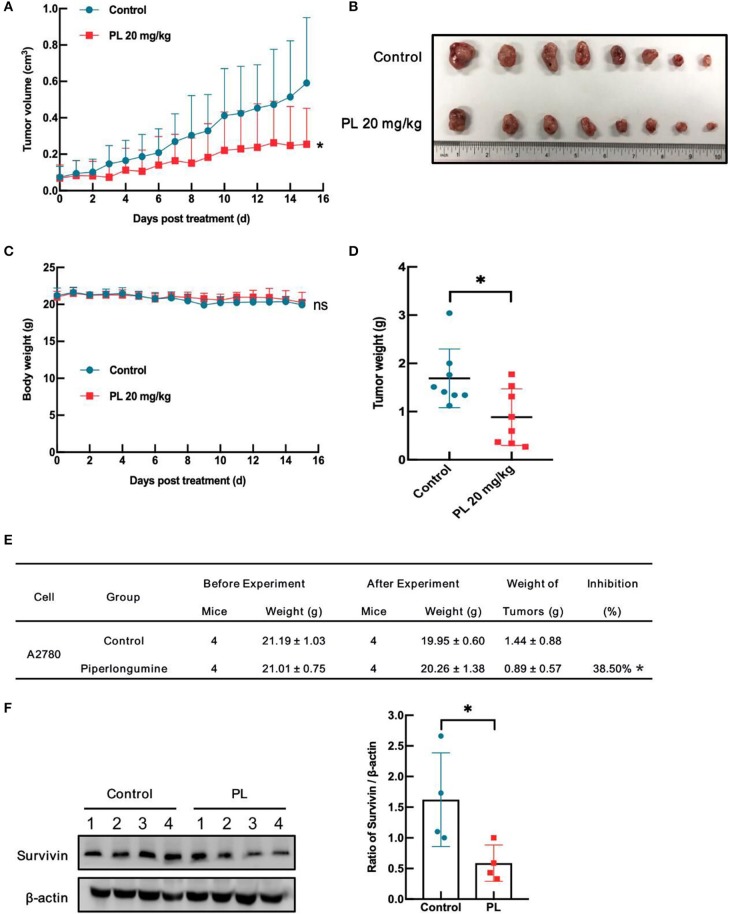
PL inhibits A2780 xenograft tumor growth and downregulates survivin *in vivo*. Each mouse was injected subcutaneously with A2780 cells (4 × 10^6^ in 100 μL of medium) under the left and right shoulders. When the subcutaneous tumors were ~0.3 cm × 0.3 cm^2^ in size, mice were randomized into two groups and received an intraperitoneal injection of the vehicle alone (0.5% methylcellulose) or PL (20 mg/kg) every day. The body weight and tumor volume were recorded. After the experiment, the mice were anesthetized, and tumor tissue was excised from the mice and weighed. The tumor volume **(A)**, original tumors **(B)**, body weight **(C)**, tumor weight **(D)**, summary data **(E)**, and Western blot analysis of survivin **(F)** were shown. β-actin served as a loading control. The band quantification was performed using ImageJ software. ^*^P < 0.05 vs. corresponding control.

To evaluate the antitumor effects of PL *in vivo*, a xenografts model of A2780 cells in BALB/c mice was conducted. Intraperitoneal injection of PL at doses of 20 mg/kg for 15 days decreased A2780 tumors weight as well as the tumor volume vs. vehicle control (Figures 6A,B,E). Notably, PL administration was endurable without severe weight loss (Figures 6C,D). Additionally, immunoblotting analyses of the tumor tissues showed that survivin was significantly downregulated after PL exposure for 15 days (Figure 6F), which was in accordance with the results *in vitro*. In short, these data indicated that PL suppresses the xenograft tumor growth *in vivo* accompanied by a decreased survivin level.

## Discussion

Because of the advanced stage at the time of diagnosis (80%) and a high recurrence rate (70–80%), human ovarian cancer surpassing other gynecological cancers lies in the fifth-leading cause of cancer-related deaths. The first-line treatment for it is the tumor cytoreductive surgery combined with platinum-taxane chemotherapy (25, 26) or neoadjuvant chemotherapy followed by debulking surgery (27). Although ~80% of patients will have a response to the frontline therapies, 25% of women within 6 months will experience resistant cancer recurrence, and the overall 5-year survival rate ranges between 30 and 40% on a global scale (1). Thus, the explore of applicable treatment drugs remains a crucial goal for carrying out a better outcome.

Owing to the abnormal expression of anti-apoptotic proteins resulting in antitumor treatments less effective or ineffective, the reactivation of cell apoptosis becomes a potential therapy to conquer antitumor drug resistance. Survivin, the smallest member of IAP proteins, participates in the inhibition of apoptosis, affects the proper process of mitosis and promotes angiogenesis or even DNA repair (28). Generally, abnormal expression of survivin is associated with decreased apoptosis, increased tumor recurrence, poor prognosis, and high chemoresistance in human ovarian cancer (29, 30). Downregulating the expression of survivin elevated the sensitivity of ovarian cancer to chemotherapy and promoted apoptosis (7, 31). Thus, elucidating the regulatory mechanisms of survivin protein expression in cancers may propose mechanistic clues to future therapeutic strategies (32, 33). In general, survivin combines with XIAP to indirectly suppress the function of caspases. XAF1 binds to XIAP resulting in the activation of ubiquitin-protein isopeptide ligase (E3) activity of XIAP, then promotes survivin ubiquitination and degradation (34). Conversely, the activation of ERK leads to the combination obstruction between survivin and the XIAP-XAF1 E3 ligase complex, which exactly stabilizes survivin proteins (35). The proapoptotic F-box protein FBXL7 acting as a ubiquitin E3 ligase interacts with Glu-126 to modulate the polyubiquitylation of survivin and thereby enhance its proteasomal degradation (36). Nonetheless, aurora kinase A (AURKA) restricts survivin to combine with FBXL7 E3 ubiquitin ligase through the negative regulation on FOXP1-FBXL7 axis, which upregulates the survivin expression (37). lncRNA LINC00473 (LNC473) remarkably suppresses the ubiquitination of survivin through recruiting deubiquitinase USP9X and then enhanced the stability of survivin (38). The Culling 9 (CUL9) is a putative E3 ligase and deplete the protein abundance of survivin via enhancing its ubiquitylation (39). Additionally, it has been reported that the ubiquitin-like-modifier proteins FAT10 (40), the heat shock protein (HSP90) (41) and CSN5/JAB1 (42) directly bind to survivin and therefore are resistant to ubiquitin-proteasome degradation. Therefore, the therapeutic regiment targeting the survivin axis is pushing the door of ovarian cancer. Such treatments include regiments that target transcription or post-translational level, vaccines based on cytotoxic activities of immune-cells and gene therapy methods that suppress survivin's function (43).

Piperlongumine, a biologically active alkaloid/amide isolated from peppers, has selectively excellent ability to inhibit tumor cells via the induction of oxidative stress and genotoxicity, as well as the significant oral bioavailability in mice burden tumor without unbearable systemic toxicity (44). We have previously reported that exposure of ovarian cancer cells to PL selectively suppressed cell viability in a concentration- and time-dependent manner (11). We deeply take an insight into the underlying mechanism of PL-induced apoptosis in ovarian cancer and find that survivin plays a vital role in the regulation of PL-mediated apoptosis in this study. Here, we firstly elucidated that PL induced apoptosis as well as the rapid depletion of survivin in ovarian cancer *in vitro* and *vivo*. Furthermore, overexpression of survivin raised the survival rate of ovarian cancer cells, implying that PL induced apoptosis of ovarian cancer via inhibiting survivin expression. In addition, our data suggested that the levels of survivin mRNA stayed unchanged under PL administration, indicating that PL induced depletion of survivin at the non-transcriptional level. This notion may lead to a rational concern about the strategy modulating either ubiquitin-proteasome or autophagy because both of them are the two primary proteolytic mechanisms. Then, a blockage of proteasome activity reversed the reduction of survivin by PL, as compared with keeping stable under autophagy inhibition. Otherwise, the previous studies also observed that the elevated levels of intracellular ROS induced by PL involved in the process of apoptosis in ovarian cancer. Considering that ROS-mediated signaling in cancer cells has long been thought to participate in malignant transformation, carcinogenesis initiation, and the survival of cancer cells, it is reasonable that the ROS-dependent pathway may take part in the regulation of survivin (45). In agreement with this notion, we observed that the apoptosis and the depletion of survivin under PL were reversed by NAC.

As all the data we assembled suggest, it is reasonable to regard the ROS-mediated proteasome-dependent mechanism as the major system responsible for the depletion of survivin under PL treatment. PL is on the way to transform ovarian cancer patients' outcomes and looking for finding its place in ovarian cancer therapeutic strategies.

## Data Availability Statement

All datasets generated for this study are included in the article/supplementary material.

## Ethics Statement

The animal study was reviewed and approved by the ethics committee of Jinan University.

## Author Contributions

X-WN, L-HG, XC, ZS, and X-JY designed the experiments, performed the experiments, analyzed the data, and wrote the paper. H-HZ, P-PY, YY, Z-HX, M-NW, YL, S-TW, and KL conducted the experiments. All authors read and approved the final manuscript.

### Conflict of Interest

The authors declare that the research was conducted in the absence of any commercial or financial relationships that could be construed as a potential conflict of interest.
